# Practical Aspects of Instantaneous Magnetization Power Functions of Silicon Iron Laminations

**DOI:** 10.1007/s42835-022-01265-2

**Published:** 2022-10-17

**Authors:** Helmut Pfützner, Georgi Shilyashki, Claes Bengtsson, Emanuel Huber

**Affiliations:** 1 Institute of Biomedical Electronics, TU Wien, 1040 Vienna, Austria; 2 ViennaMagnetics GmbH, 1020 Vienna, Austria

**Keywords:** Instantaneous magnetization power, Equivalent circuits, Loss resistance, Silicon iron, Hysteresis, Eddy currents

## Abstract

Magnetic energy loss *P* of SiFe steel represents a key factor for the efficiency of soft magnetic machine cores. Traditionally, they are operated with 50 Hz (or 60 Hz), a frequency value that yields rather balanced portions of hysteresis loss and eddy current loss. In equivalent circuits of transformers, *P* tends to be represented by a magnetic power resistance *R*_M_, as a constant. For the most important case of sinusoidal induction *B* of 50 Hz, this would correspond to an instantaneous magnetization power function *p*(*t*) that is sinusoidal as well, however, with 100 Hz (or 120 Hz). On the other hand, from complex, non-linear mechanisms of hysteresis, it is obvious that *p*(*t*) should be strongly non-sinusoidal, even for exactly sinusoidal *B*(*t*). So far, almost all corresponding instantaneous investigations were restricted to calculated modelling of loss portions and transient modelling. On the other hand, for the first time, the present study was focussed on functions *p*(*t*) as measured at IEC-standardized samples of industrially relevant steel. Practical evaluations are discussed with respect to the revealed “history” of magnetization processes, as well as for product characterization. For these tasks, a novel digitized “Low-mass Single Sheet Tester” was developed that was applied for both non-oriented steel (NO) and grain-oriented steel (GO), for 50 Hz. Interpretations proved to be favoured by relating *p*(*t*) to total *P*, according to an instantaneous power ratio. As a result, both steel types revealed strongly non-sinusoidal power functions, with short durations of negative *p*. Negative *p* proved to be most pronounced for NO steel, as a measure for the onset of reversible turns of atomic moments. As a consequence, *p*(*t*) comprises strong upper harmonics of 200 Hz and even 300 Hz. Based on theoretical considerations, we split *p*(*t*) in a dissipative loss power function *p*_L_(*t*) and in a potential energy power function *p*_P_(*t*). Finally, we used *p*(*t*) to determine the corresponding power resistance *R*_M_(*t*) that proves to be a distinctly nonlinear function as well. It resembles a rectified co-sinus, also exhibiting short negative spikes that reflect the crystallographic dis-orientation of the polycrystalline material.

## Introduction

Steadily increasing interest exists for the energetic efficiency of electric machines. This includes magnetic energy losses of silicon iron laminations, as applied for soft magnetic cores of machines like transformers, generators and motors. Continuous attempts are made for technological improvements of both the machines and the applied materials, in particular for a reduction of losses. Special interest exists in no-load losses *P* for those machine types that are in continuous operation, like power transformers, the cores of which are constructed from grain oriented SiFe. On the other hand, cores of small transformers tend to be manufactured from non-oriented steel, as also applied for shunt reactors, even in cases of largest dimensions. Recently, these steels became relevant also for compact motors of electric vehicles.

Since hundred years, attempts are made to minimize losses of a given type of material, among others on the basis of loss portions. Traditionally, total losses *P* are separated into hysteresis loss and eddy current loss, including so-called excess loss. Compact mathematical expressions were established for a corresponding split-up (e.g. [1–3]). However, their applicability for modern types of materials is complicated through complex technological measures: Hysteresis loss is influenced by a high number of impact factors like chemical composition, texture, magnetostrictive features, surface structure, but also by internal mechanical stress (including forced one, through stress coatings or scribing). Similar dependencies exist for eddy current loss, which impedes an effective mathematical split-up.

All above mentioned loss terms tend to be discussed as quantities that are averaged over a period of magnetization. However, for AC operation, it is obvious that loss quantities vary as a function of time, during a period of magnetization. Actually, for power machines, such variations are assumed to be insignificant, as long as the machine is under full load. On the other hand, transformers and shunt reactors may be unloaded for long durations of time. This means that “no-load” losses become representative for the effective loading of the grid system, and also for possible retro-active effects on the latter, like the generation of ripple.

**Fig. 1:**
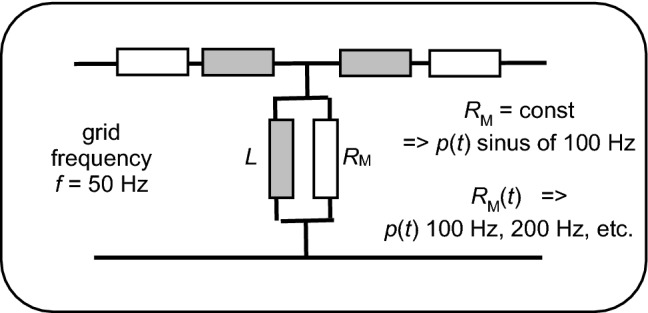
Traditional consideration of magnetic energy losses in a transformer equivalent circuit, through a magnetic power resistance *R*_M_

Since many decades, both stationary and transient performances of electric machines are modeled by means of electric equivalent circuits. In particular, circuits of transformers (Fig. 1) tend to contain a—differently entitled—magnetic power resistance *R*_M_ (frequently also *R*_o_) that considers total magnetic energy losses, i.e. the sum of hysteresis loss and eddy current loss. Though hysteresis mechanisms are of clearly non-linear nature, *R*_M_ tends to be assumed as a constant, with single exceptions, like in reference [4] that discusses an induction generator. Constant *R*_M_ is even assumed for calculations of transient problems, like that of inrush currents [5–10]. Attempts to consider the involved non-linearity are rare, like that reported in [11] where Preisach principles are introduced. As an alternative, [12] uses domain theory, however, for high-frequency materials.

A priori, increased levels of understanding can be expected from studies of instantaneous magnetization power functions *p*(*t*), i.e. of the temporal “history” of power, during a period of magnetization. However, studies of functions *p*(*t*) are discussed in literature in very rare ways. In particular, little knowledge exists about the involved temporal functions of dynamic power variations, as being typical for different practically relevant steel types.

Already in 1962, the temporal distribution of energy dissipation was addressed by Kneller [2]. Much later, references [3, 13] reported estimations of instantaneous eddy current loss power, on the basis of the derivative of induction *B*, for sinusoidal and non-sinusoidal induction *B*(*t*), respectively. Reference [14] is focused on instantaneous powers in non-linear switched circuits. Reference [15] reports instantaneous hysteresis loss and eddy current loss, as calculated numerically on the basis of the Jiles–Atherton model [16]. More recently, Petrun et al. [17, 18] reported power functions that were calculated theoretically for rather high frequencies of 1000 Hz and 400 Hz, respectively. The applied, so-called “parametric magneto-dynamic (PMD) model” describes magnetization dynamics of a sheet that is virtually separated into several equally thick slices, individual power functions being calculated for the latter.

The above mentioned studies were focused on the modelling of dynamic processes in mathematical ways. On the other hand, the here reported study is aimed on the characterization of different types of core steel by means of the experimental measurement of its individual power functions. As a pre-requisite for exact measurements, a specific type of digitized Single Sheet Tester was developed. As a main task, the latter was applied for tests on samples of grain-oriented (GO) steel and non-oriented (NO) steel, respectively, and the resulting magnetization power functions *p*(*t*) were evaluated and discussed in comparative ways.

## Definitions and State of the Art

As it was proved in [19], time-averaged total losses (in W/kg) of an AC magnetized steel sample can be expressed in very simple ways as

1 $$P = 1/(\rho T) \cdot \int\limits_{T} {H(t) \cdot {\text{d}}B/{\text{d}}t} \cdot {\text{d}}t = 1/T \cdot \int\limits_{T} {p(t) \cdot {\text{d}}t,}$$

with *ρ* the density of sample and *T* the length of period of magnetization. The equation yields total losses under the condition that a flat sample sheet is magnetized in axial direction *x* and that we define *H*(*t*) as the field of the sample surface and *B*(*t*) as the induction as averaged over the samples cross section. A further condition is that *H*(*t*) and *B*(*t*) do not vary along the axis *x*. The equation contains the instantaneous magnetization power function in W/kg as the term

2 $$p\left( t \right) = {1}/\rho \, \, H\left( t \right)\, {\text{d}}B/{\text{d}}t$$

which is averaged over the cross section.

For an assessment of the corresponding state of the art, we performed an intensive search of literature. In a quite surprising way, the latter revealed that measured power functions *p*(*t*) are restricted to reference [20] as a single study. It contains functions for both NO-steel and GO-steel in comparison to dynamic magnetization loops. But the results indicate difficulties of temporal triggering of specific events that are relevant for the physical interpretation of the “history” of magnetization processes which however are not addresses in [20].

On the other hand, the here reported project [21] seems to be a first approach for exact measurements of power functions on practically representative, large IEC-standardized (500 mm × 500 mm sized) samples of material, with the aim of its characterization through time patterns of magnetization.

In our so far work, we demonstrated in [22] for GO-steel that power functions may favor the design of loss testers since indicating that dissipation is concentrated in instants of low induction levels. Further, in [23], we reported a complex function for NO-steel to illustrate the need to characterize different materials through measurements instead of calculation that cannot consider modern technologies of steel production.

Keeping the latter in mind, we restrict all aspects of the present paper to data that is established in experimental ways. As a first step, here we concentrate on the industrially most relevant case of *f* = 50 Hz (being relevant also for 60 Hz), in connection with standard levels of peak induction *B*_PEAK_.

## Methodology

### Experimental Preconditions

A priori, studies of instantaneous magnetization power functions *p*(*t*) are handicapped by the fact that standardized loss testers [24, 25] are designed for the determination of total losses *P* that are averaged over the period *T* of magnetization by means of the Watt-metric principle. The latter does not offer an exact determination of the magnetic field strength *H*(*t*), as it is needed for an effective assessment of *p*(*t*). Both standardized testers, the Single Sheet Tester (SST; [24]) and the Epstein Tester (ET; [25]) derive the field in indirect ways from the magnetization current. The latter reflects the excitation of the whole magnetic circuit that is strongly inhomogeneous, for both types of tester.

As an alternative, [20] reported the use of a homogeneous wound core. However, the latter involves mechanical stress from the samples curvature, as a source of errors, at least for grain oriented steel. A more promising option is given by apparatuses with tangential field coils (e.g. [19, 26]). However, so far, their availability is strongly restricted, apart from drawbacks that result from the usually very small sample size, that lacks industrial representativeness.

### Low-Mass Single Sheet Tester

For the routine detection of averaged total losses *P*, it can be assumed that the most effective so far available tool is given by the already mentioned standardized Single Sheet Tester (SST) [24]. It is highly robust and offers tests of very good reproducibility. On the other hand, it proves to be ineffective for measurements of basic physical nature, as in the here given case. This is mainly due to the following two reasons:

(i) As already mentioned, the SST does not allow for an exact determination of the course of time *H*(*t*) of magnetic field strength.
(ii) The sample is enclosed by a massive double yoke of hundreds of kilogram mass, a design that prevents access to the sample, as it is needed for flexible experimental work.

As a tool for versatile analyses of dynamic features of losses up to 500 Hz, we developed a “Low-mass Single Sheet Tester” (L-SST) that avoids both drawbacks by a replacement of the current method by the already mentioned field method [19, 26]. A double yoke is not needed. Apart from a distinct mass reduction, this means that the field *H*(*t*) of the sample surface can easily be detected by a tangential field coil (H-coil).

**Fig. 2:**
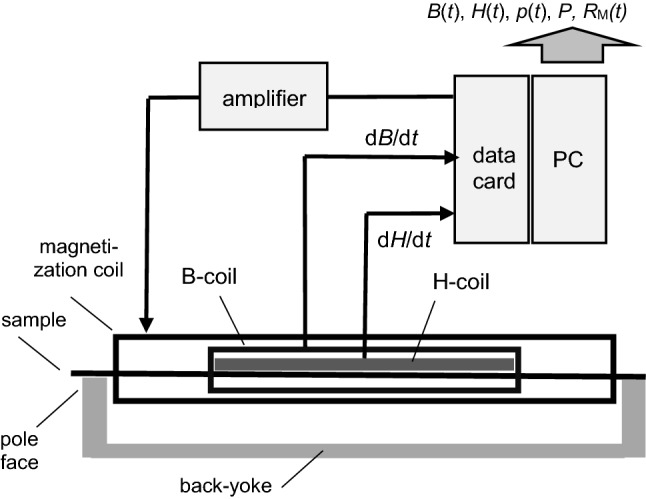
Low-mass Single Sheet Tester for a physically consistent determination of magnetic energy losses, including instantaneous characteristics

Figure 2 shows the principle of L-SST in schematic ways. In accordance to IEC standards, the sample is given by a quadratic single sheet of 500 mm × 500 mm size. For the detection of field, the H-coil is arranged directly over the sample surface. With a size of 320 mm × 480 mm, it covers the majority of the sample area. With a thickness of less than 2 mm, it effectively detects in just 1 mm from the sample surface. This makes errors through demagnetizing field components negligible. According to standards, the pole faces of the L-SST show a width of 25 mm, for effective coupling between sample and yoke. However, for mass reduction, the back-flux is carried by a single yoke the thickness of which is reduced to about 15 mm. This design yields a very distinct reduction of mass, down to about 80 kg.

The induction *B* is detected in physically consistent ways by means of a B-coil of 320 mm length, in connection with digital air flux correction. Losses *P* are calculated from *H*(*t*) and *B*(*t*) on the basis of Poynting Theorem, under the condition of 1-D energy flow, with Poynting vectors perpendicular to the sample area. This is attained by the fact that the detection of both quantities is restricted to the samples central region of 320 mm length. Practically, the latter exhibits homogeneous axial flux, as confirmed by tangential induction sensors [27] that were shifted along the sample surface. Under these conditions, losses of the sample end regions are without relevance for the evaluated loss result *P*. This is also valid for the contact regions between sample and yoke, and for the yoke itself. Taking advantage from these conditions, the yoke could be constructed from non-oriented SiFe, in rather compact ways.

The tester allowed for a direct determination of both total time-averaged losses *P* and instantaneous power functions *p*(*t*), both in W/kg.

## Magnetic Power Functions and its Portions

By means of the above described apparatus, we measured instantaneous magnetization power functions according to (2), i.e. *p*(*t*) = 1/*ρ* *H*(*t*) · d*B*/d*t*.

**Fig. 3:**
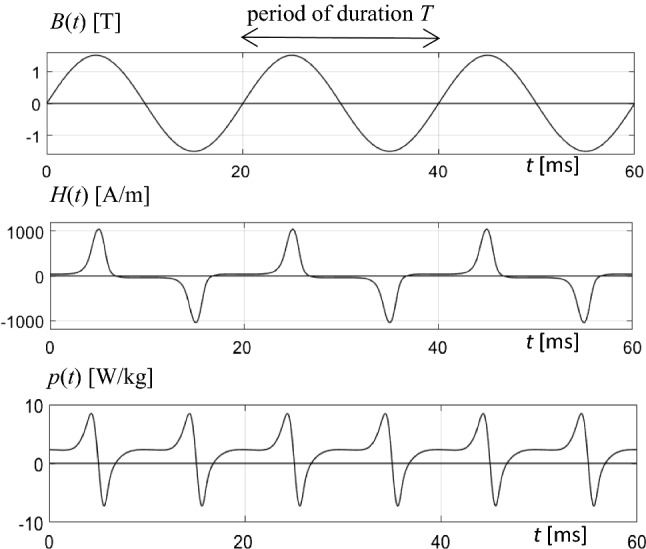
Example for courses of time of instantaneous quantities, for three periods of magnetization with 50 Hz. Induction *B*(*t*), magnetic field strength *H*(*t*) and magnetization power *p*(*t*)

For a global illustration of observed temporal tendencies, Fig. 3 shows a typical example of a function *p*(*t*), for three periods of magnetization, according to sinusoidal induction *B*(*t*). As it is well known, for the technically most relevant cases, the corresponding magnetic field *H*(*t*) is characterized by a positive spike, followed by a negative one. The almost constant plateaus between the spikes reflect the dynamic coercivity, in approximation. The signal pattern tends to be similar for different materials, while the spike intensities may differ in very strong ways. Symmetry arises for lacking DC-bias, as it was given in the present study.

The function of instantaneous power *p*(*t*) is characterized by doubled fundamental frequency, that is 100 Hz in the here given case of magnetization with *f* = 50 Hz. Per period, the function shows two positive maxima of strongly varying shape—according to the further down results. On the other hand, two negative minima arise with restricted intensity and spike-like shape.

According to (2), the instantaneous power function *p*(*t*) is determined by the magnetic field strength *H*(*t*) as arising on the surfaces of material and by the time derivative d*B*/d*t* averaged over the materials cross section. As it is well known, the quantity *P* expresses time-averaged total losses, as resulting from energy dissipation into heat. On the other hand, we express *p*(*t*) as the sum of two instantaneous power components, according to

3 $$p\left( t \right) = p_{{\text{L}}} \left( t \right) + p_{{\text{P}}} \left( t \right)$$

Here *p*_L_(*t*) is the loss power that represents the instantaneous dissipation due to Bloch wall interactions and eddy current effects. On the other hand, *p*_P_(*t*) is of non-dissipative nature, expressing the potential energy power, as it is needed for turns of atomic moments. In Fig. 3, the positive and negative peaks of *p* are mainly due to this mechanism.

Theoretically, a magnetization power function *p*(*t*) can be interpreted in an analogous way to a dynamic magnetization loop, as closer discussed in [19]. However, the power pattern shows the advantage that it offers the temporal development in a transparent, straight-forward, linear way that favors interpretations. On the other hand, the fact that the instantaneous power exhibits two portions complicates routine interpretations of measured functions. However, as described in Sect. 6, it proved to be possible to separate a power function into the two portions of (3), at least in approximate ways.

We performed measurements for several types of material, as it will be described elsewhere, also as a function of frequency *f*. On the other hand, the present paper is concentrated on *f* = 50 Hz, in connection with the following two types of material:

NO-sample—non-oriented, thickness 300 µm,

*P* = 1.83 W/kg

for an induction peak value *B*_PEAK_ = 1.5 T

GO-sample—grain oriented, thickness 218 µm,

*P* = 1.18 W/kg for *B*_PEAK_ = 1.7 T

Measurements were performed for the above mentioned values of peak induction, considering that they represent the key inductions for industrial application of the tested steel types.

## Non-oriented Material

**Fig. 4:**
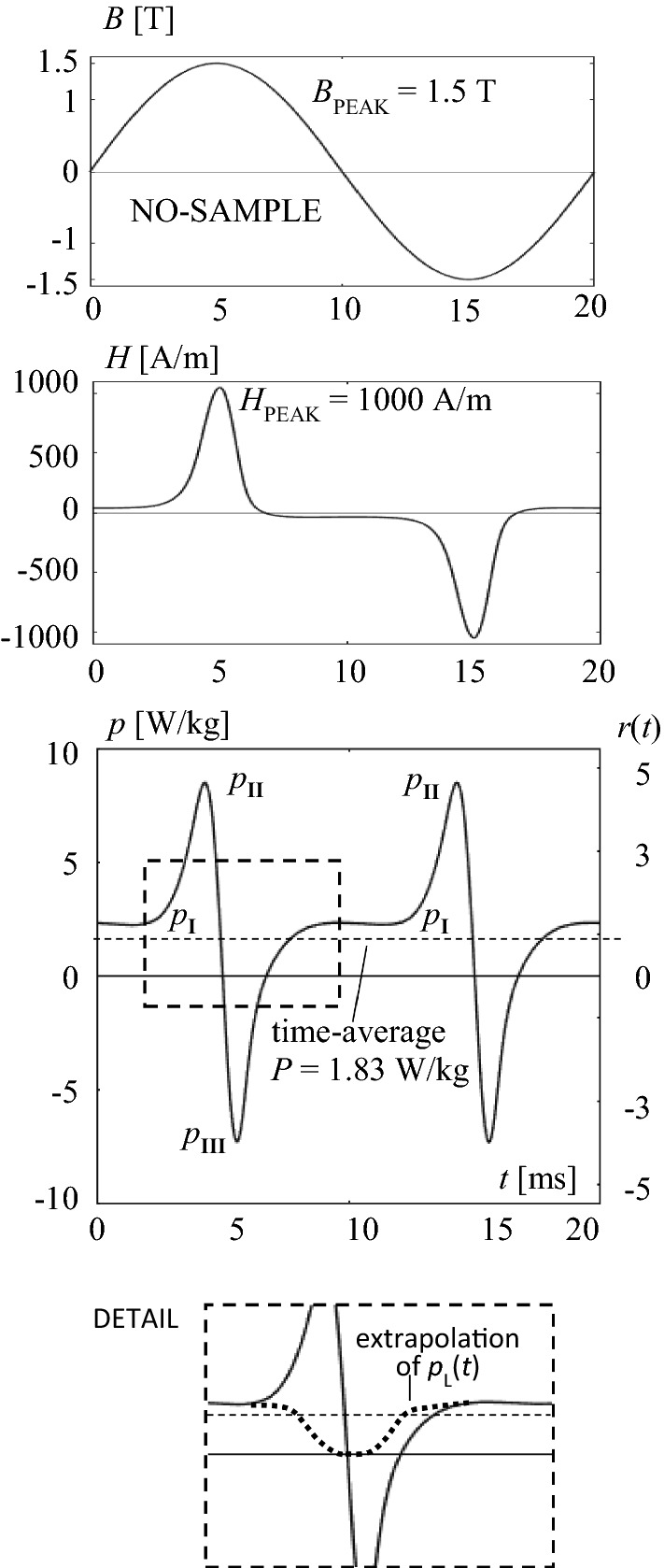
Instantaneous quantities for the non-oriented NO-sample, for one period. Induction *B*(*t*), magnetic field strength *H*(*t*) and magnetization power *p*(*t*). Detail: Dashed curve as a hypothetical extrapolation of *p*_L_(*t*) in II and III

For the NO-material, Fig. 4 shows a result of a power function *p*(*t*) for a single period. The NO-sample was magnetized in exactly sinusoidal ways, with a peak induction of *B*_PEAK_ = 1.5 T. The graph considers a period of 20 ms duration, according to considerably high time-averaged total losses of *P* = 1.83 W/kg.

The corresponding profile of instantaneous magnetization power shows a long-time plateau of *p*_I_ = 2.1 W/kg intensity, for the time interval of low induction intensity. On the other hand, for the instant *t* = 5 ms of maximum induction, we find *p* = 0. Shortly before this instant, we find an intensive positive, spike-like peak of *p*_II_ = 8.5 W/kg, shortly afterwards an almost as intensive negative peak of *p*_III_ = − 7.3 W/kg.

A physical interpretation is enabled by generally accepted theories of ferromagnetism. Within the graph *p*(*t*), the halve period of magnetization is separated into three intervals:

### Interval I (up to ca. *t* = 3 ms, and again from 7 to 13 ms)

This interval is characterized by moderate values of induction *B*. It includes the instant *t* = 10 ms of *B* = 0, that corresponds to maximum d*B*/d*t*. Here, the power shows *p*_I_ = 2.1 W/kg which is more than 10% above total losses of 1.83 W/kg. A maximum amount of main Bloch walls is given. They move with high velocities, as a reason for strong eddy currents (ECs), as the basic source of energy dissipation, on three levels:

(i) Global ECs round the axis of material yield high contributions to “classical” eddy current losses *P*_E,C_.
(ii) Local ECs round the moving walls contribute to “excess” eddy current losses *P*_E,A_, however, for the here given NO material, with weak intensity.
(iii) Sub-microscopic ECs round activated pinning centers, can be assumed to be the main mechanism for the loss category that tends to be nominated as “hysteresis losses” *P*_H_. In the case of NO material, they tend to be strong, since also the boundaries of the typically very small grains contribute to pinning.

With the above, intervals I reflect merely dissipative power *p*_L_. That is, they yield the main contributions to total losses *P*. This corresponds to the fact that the instantaneous power level *p*_I_ exceeds *P* by the already mentioned 11%, for the here given frequency *f* = 50 Hz. For higher *f*, the level of values *p*_I_ would be further increased.

### Interval II (from *t* = 13 ms up to 15 ms)

This interval concerns approaching saturation, however, with *B*_PEAK_ = 1.5 T, in restricted ways. Bloch walls get annihilated, at least in well oriented grains. This means that all above mentioned mechanisms i…iii loose relevance, according to very weak energy dissipation. In spite of the latter, the instantaneous power exhibits a pronounced, spike-like maximum *p*_II_ that indicates a strong increase of potential energy, according to a dominant portion *p*_P_. The peak reflects the over-all amount of reversible turns of atomic moments.

### Interval border II/III (*t* = 15 ms)

This instant is given by maximum induction *B* = *B*_PEAK_. As it is evident—but frequently neglected in discussions—energy dissipation is zero here, due to d*B*/d*t* = 0. The velocity of (residual) Bloch walls is zero, corresponding to lacking eddy currents and lacking hysteretic interactions with pinning centers. This yields zero loss power *p*_L_ = 0, and also zero potential energy power *p*_P_ = 0, since turns of moments are not supported by the needed field. On the other hand, they arise left and right of the border. There, also weak loss power *p*_L_ is to be expected, an estimation following from a hypothetical extrapolation of *p*_L_(*t*) in Interval I (see detail in Fig. 4).

At this point, it should be noted that the simple above considerations are based on the Maxwell equations, as a macroscopic model for magnetic phenomena. In fact, the interval close to maximum induction tends to be governed by reversible mechanisms, the closer discussion of which needs thermo-dynamic modelling, under adiabatic conditions, as described in earlier literature like [1–3].

### Interval III (from *t* = 15 ms up to 17 ms)

This interval concerns reversal re-turns of magnetic moments as well as re-nucleation of walls. The power shows a negative peak of intensity *p*_III_ that indicates the sudden fall of potential energy, according to *p*_P_. The rather small intensity difference between *p*_II_ as the positive peak intensity and *p*_III_—as the negative one confirms that the interval II contributes to total losses *P* in weak ways. This is in contrast to theories that expect distinct hysteresis in instants of high *B* due to annihilation and nucleation of Bloch walls.

## Grain-Oriented Material

**Fig. 5:**
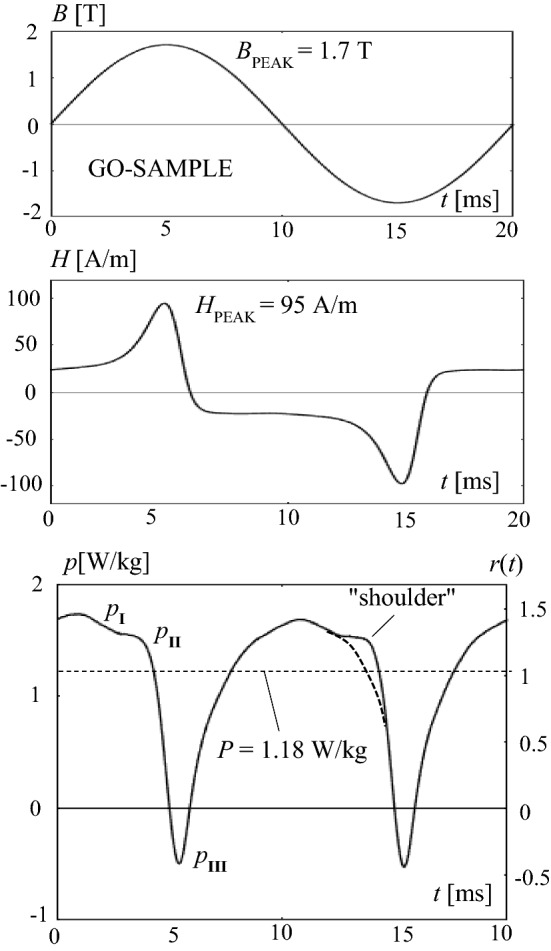
Results for the grain-oriented GO-sample, for *B*_PEAK_ = 1.7 T. Induction *B*(*t*), magnetic field strength *H*(*t*), and magnetization power *p*(*t*)

As it is illustrated by Fig. 5 for a peak induction *B*_PEAK_ = 1.7 T, the GO-sample proved to yield a substantially different temporal power profile. The signal is characterized by strong asymmetry, with an un-even plateau of about *p*_I_ = 1.8 W/kg maximum intensity. While the positive maximum of *p*_II_ = 1.6 W/kg is visible only in hidden ways, the negative one of *p*_III_ = − 0.5 W/kg is pronounced, but much weaker than that for the NO case.

For an interpretation, the power function of Fig. 5 reveals from the first glance that a substantially different performance is given in comparison to NO-material. In particular, the positive peak of Interval II is missing here. In some detail, the function reveals the following:

### Interval I (up to ca. 3 ms, and from *t* = 7 ms up to 13 ms)

Considering the changed scale, we see that instantaneous losses are much weaker than for NO steel. Lower losses can be attributed mainly to lower hysteresis losses, in the course of an ordered structure of both grains and domains. With *p*_I_ = 1.8 W/kg, the instantaneous power intensity exceeds averaged losses *P* = 1.18 W/kg by about 50%. This increase is much stronger than in the NO case, due to a temporal concentration of energy dissipation.

### Interval II (from *t* = 13 ms up to 15 ms)

The positive peak appears in a hidden way as a kind of shoulder with *p*_II_ = 1.5 W/kg. Its low intensity indicates a high degree of grain orientation of the tested material. At least up to the given peak induction *B*_P_ = 1.7 T, turns of magnetic moments prove to be insignificant. The temporal function *p*_L_(*t*) can be assumed to follow the dotted line (analogous to that of Fig. 4).

### Interval III (from *t* = 15 ms up to 17 ms)

The low intensity *p*_III_ =—0.4 W/kg (instead of—7.3 W/kg for NO) confirms the high degree of texture. Even lower peak intensity proves to arise for highly GO materials, due to a quasi single-crystal performance. The negative peak balances the positive shoulder with equal areas.

## General Model for Power Separation

**Fig. 6:**
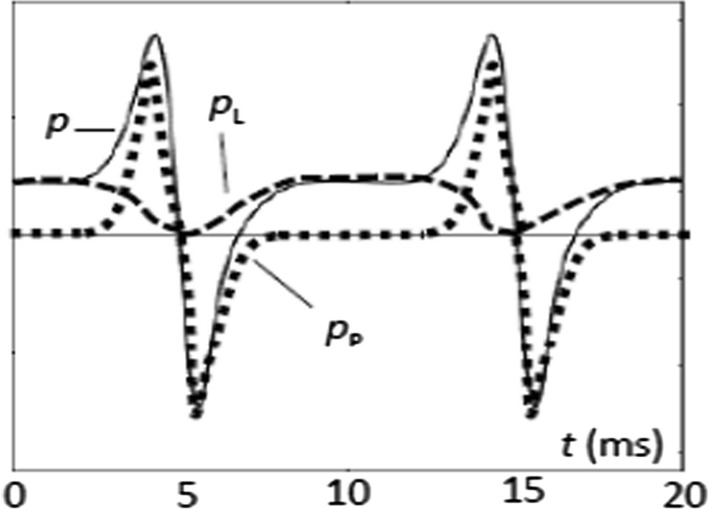
Total power function *p*(*t*) as the sum of a (dashed) hump-like loss power function *p*_L_(*t*) and a (dotted) spike-like potential energy power function *p*_P_(*t*) that consistes of two elements

Comparing the above results with additional ones of other steel types, we conclude that the power function *p*(*t*) can be generally interpreted by the summing-up the two components as given by (3), in the following way:

(i) The loss power *p*_L_(*t*) is represented as a hump-like function (dotted lines in Fig. 6), a hump showing a duration of the halve period length. For 50 Hz, it starts with zero at instant *t* = 5 ms, reaches its maximum shortly after 10 ms and ends with zero at 15 ms. The hump heigth *p*_I_ corresponds with the maximum of instantaneous losses.
(ii) The potential energy power *p*_P_(*t*) is represented by two spikes (dashed lines in Fig. 6), a positive one prior to the end of hump and a negative one at the start of the following hump. The maximum is a measure for the amount of turns of magnetic moments, as arising for the involved peak value *B*_PEAK_ of induction.

In approximation, the spikes are super-imposed to the humps in additive ways. Weak further impact seems to result from hysteretic effects that need further study. However, approximative separations are possible, in accordance also with the dashed curves in Figs. 4 and 5.

## Determination of Dynamic Loss Resistance

**Fig. 7:**
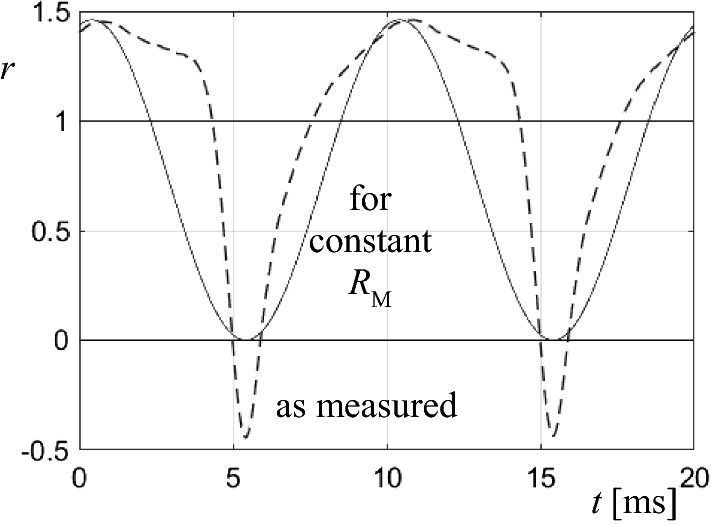
Comparison of related power patterns *r* as a function of time *t* for modelling with constant *R*_M_ (full curve) and an experimental result (dashed curve), for the GO-sample

Finally, the last task of the present study was to apply the magnetization power functions *p*(*t*) for the evaluation of the corresponding dynamic loss resistances *R*_M_(*t*). According to Fig. 1, in electrical equivalent circuits, magnetic losses tend to be considered by a quantity *R*_M_ that is assumed as a constant. The full curve of Fig. 7 shows the corresponding power function related to total losses, as a related power

4 $$r\left( t \right) = p\left( t \right)/P$$

For the no-load case with sinusoidal magnetization with 50 Hz, this would mean that the function *p*(*t*) describes a sinus with 100 Hz of mean value *r*_MEAN_ = *1*. This would corresponds to *r*_I_ = 2 and *r*_III_ = 0.

From the first glance, it is clear that the sinus curve is not valid for our two cases of material, even not in rough approximation. The case that is closer to the model of constant *R*_M_ is the GO-case, as illustrated again in Fig. 7 through a dashed curve, in comparison to the sinus function.

**Fig. 8:**
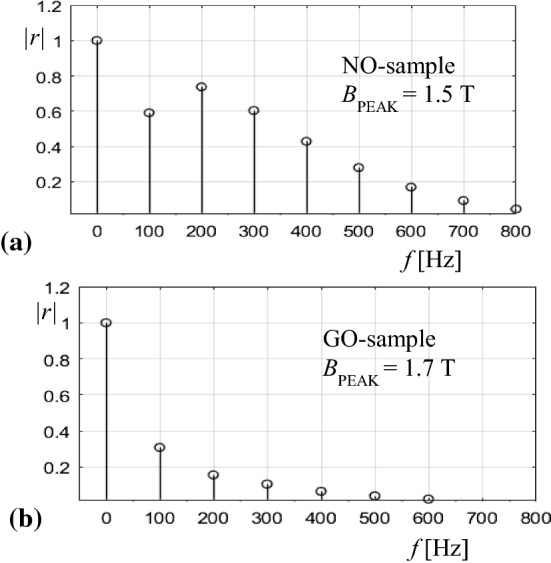
Fourier spectra for the measured related functions *r*(*t*). **a** Non-oriented NO-sample. **b** Grain-oriented GO-sample

For an illustration of tendencies, Fig. 8 shows the spectral lines of *r*(*t*) for the two investigated types of material. The non-oriented NO-sample exhibits strongest harmonics, the lines for 200 Hz and 300 Hz even exceeding that of 100 Hz. This can be interpreted by a dominant role of hysteresis. The GO-sample shows much weaker upper harmonics.

Finally, the determination of the corresponding temporal changes *R*_M_(*t*) was based on the equivalent circuit Fig. 1 for the unloaded state, using the expression

5 $$p\left( t \right) = u\left( t \right)^{2} /R_{{\text{M}}} \left( t \right).$$

We introduce

6 $$u\left( t \right) = F \cdot {\text{d}}B/{\text{d}}t$$

with *F* as a proportional factor that depends on the geometry of the considered soft magnetic core. This yields the instantaneous loss resistance as

7 $$R_{{\text{M}}} \left( t \right) = F^{{2}} \cdot \left( {{\text{d}}B/{\text{d}}t} \right)^{2} /p\left( t \right).$$

**Fig. 9:**
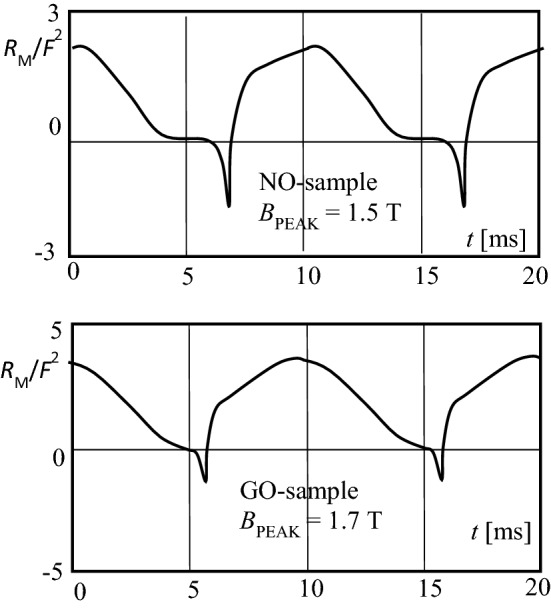
Magnetic loss resistance *R*_M_ (with *F*^2^ as a geometry factor) as a function of time *t*. **a** Non-oriented NO-sample. **b** Grain oriented GO-sample. *Notice*: Effects from division through zero are corrected

Figure 9 shows the resulting functions *R*_M_(*t*) for one period, for the two types of material. As to be expected, *R*_M_ varies with time in distinct ways. It shows maximum values for time intervals of strong induction changes. In consistent ways to instantaneous power values, *R*_M_ is negative in the remanent intervals. Stronger variations arise for the non-oriented material, due to the dominance of hysteresis mechanisms.

Contrary to very distinct differences between power functions *p*(*t*), the corresponding resistance functions *R*_M_(*t*) exhibit similar patterns, resembling rectified co-sinus functions, in rough approximation. Short negative spikes are pronounced for the NO-sample.

## Discussion

A very detailed search of literature proved that temporal evaluations of measured power functions have not been performed before. In particular, it cannot be expected that major text books on magnetism may contain any corresponding data. This is also valid for more recent reviews on transformers like [28, 29].

The clear reason for the above is that conventional electronics did not offer the here needed dynamic resolution of data processing. For an illustration, the advanced data acquisition card according to Fig. 2 enables simple A/D conversion (of 200 kHz sampling rate) of field and induction. However, their seemingly simple link according to (2) proves to need two-period multiplexing, due to electro-static loading processes [30]. In particular, this means that power functions of non-oriented steels (Figs. 4, 9a) still represent a challenge, with respect to sharp spikes from spin orientation processes.

However, the results are consistent with theoretical expectations. We conclude that power functions *p*(*t*) may prove as novel tools for simple interpretations of magnetization mechanisms, of individual types of SiFe steel products.

In particular, the functions are attractive for the comparison of different products. However, this comparison proves to be complicated by the fact that the involved orders of *p* may differ in too strong ways. For easier comparisons of different types of material, we evaluated related functions *r*(*t*) = *p*(*t*)/*P*, according to (4). In Figs. 4 and 5, they are offered by a second scale at the right side. In an analogous way, we define related characteristics like *r*_I_ = *p*_I_/*P*, *p*_II_/*P* and *p*_III_/*P*. These characteristics promise compact and rapid comparisons of different steel types.

For the investigated two types of material, some numerical characteristics are summarized in Table 1. The evaluated data reflect in clear ways the strong dynamics of power functions of the NO-sample and the low dynamics for GO. The very strong variations of characteristics indicate high potential for the identification of different technologies of steel production.

**Table 1:** Characteristics for different steel types (instantaneous power ratios *r*)

Characteristic	Symbol	NO-sample	GO-sample
Related power plateau	*r*_I_	1.2	1.5
Related positive peak	*r*_II_	4.6	1.4
Related negative peak	*r*_III_	− 4.0	− 0.4
Peak ratio	*r*_III_/*r*_II_	− 0.87	− 0.29

Further work will be aimed on attempts to utilize such characteristics for the estimation of the ratio of averaged loss components, with the advantage of minimum expenditure of work. The challenge is to collect a maximum of information from a power function that results from a single period of magnetization. Attempts will be made to establish corresponding quantitative algorithms. Further tasks will be to study power functions for magnetization in transverse direction, as well as for rotational magnetization—at least in approximate ways.

With respect to time functions of loss resistance *R*_M_(*t*), we assume that they can easily be integrated in equivalent circuits in approximated ways as a rectified co-sinus pattern. At this point, it should be remind to the fact that stationary sinusoidal induction was assumed throughout the present study. A possible method for the modelling of transient problems may be to adapt data in iterative ways.

## Main Conclusions

Traditionally, magnetic losses are considered in electric equivalent circuits like that of transformers by a loss resistance that is assumed as a constant quantity. Starting out from time functions of magnetization power, the here reported modelling reveals that this assumption cannot be expected to be effective, at least not for the here considered low-frequency case of 50 Hz.

The main conclusions of the present study are the following:

1. Even in the here investigated simple case of non-distorted, sinusoidal induction, instantaneous power functions are characterized by very distinct distortion, during a period of magnetization.
2. Positive power intervals are partly counter-balanced by spike-like negative portions.
3. Most pronounced distortion of power functions is indicated for non-oriented silicon steel, due to dominant effects of hysteresis, and of crystalline disorientation.
4. Compared to discussions of dynamic magnetic loops, the interpretation of loss mechanisms by means of power functions shows the advantage of a clear linear temporal history of magnetization.
5. For electric equivalent circuits of machines or apparatuses, power functions allow for the determination of non-linear loss resistance functions the time pattern of which resembles a rectified co-sinus function, that is interrupted by short, spike-like negative intervals.
